# Supplementary material for: Dichotomous Effects of Glypican-4 on Cancer Progression and Its Crosstalk with Oncogenes
Source: Int J Mol Sci. 2024 Apr 2;25(7):3945. doi: 10.3390/ijms25073945 (PMC11012302; doi:10.3390/ijms25073945)
Supplement: Supplementary file 1 [file ijms-25-03945-s001.zip › ijms-2901394-supplementary.pdf]

Supplementary Table S1: List of TCGA study abbreviations

Study Abbreviation	Study Name
ACC	Adrenocortical carcinoma
BLCA	Bladder Urothelial Carcinoma
BRCA	Breast invasive carcinoma
CESC	Cervical squamous cell carcinoma and endocervical adenocarcinoma
CHOL	Cholangiocarcinoma
COAD	Colon adenocarcinoma
DLBC	Lymphoid Neoplasm Diffuse Large B-cell Lymphoma
ESCA	Esophageal carcinoma
GBM	Glioblastoma multiforme
HNSC	Head and Neck squamous cell carcinoma
KICH	Kidney Chromophobe
KIRC	Kidney renal clear cell carcinoma
KIRP	Kidney renal papillary cell carcinoma
LAML	Acute Myeloid Leukemia
LGG	Brain Lower Grade Glioma
LIHC	Liver hepatocellular carcinoma
LUAD	Lung adenocarcinoma
LUSC	Lung squamous cell carcinoma
MESO	Mesothelioma
OV	Ovarian serous cystadenocarcinoma
PAAD	Pancreatic adenocarcinoma
PCPG	Pheochromocytoma and Paraganglioma
PRAD	Prostate adenocarcinoma
READ	Rectum adenocarcinoma
SARC	Sarcoma
SKCM	Skin Cutaneous Melanoma
STAD	Stomach adenocarcinoma
TGCT	Testicular Germ Cell Tumors
THCA	Thyroid carcinoma
THYM	Thymoma
UCEC	Uterine Corpus Endometrial Carcinoma
UCS	Uterine Carcinosarcoma
UVM	Uveal Melanoma

Supplementary Table S2: List of genes with divergent gene expression profiles between TCGA-LUAD and TCGA-GBM subjects

	Term	Overlap	P.value	Adjusted.	Old.P.valu	Old.Adjus	Odds.Rati	Combinec	Genes	database
1	Hypoxia	2/200	0,007218	0,021654	0	0	18,17172	89,60771	CA12;STC1	MSigDB_Hallmark_2020
2	Estrogen Response Early	2/200	0,007218	0,021654	0	0	18,17172	89,60771	CA12;PTGI	MSigDB_Hallmark_2020
3	Estrogen Response Late	2/200	0,007218	0,021654	0	0	18,17172	89,60771	CA12;PTGI	MSigDB_Hallmark_2020
4	Epithelial Mesenchymal Transition	2/200	0,007218	0,021654	0	0	18,17172	89,60771	ITGA5;DKI	MSigDB_Hallmark_2020
5	Fibronectin Matrix Formation R-HSA-1566977	1/6	0,003894	0,046096	0	0	333,0333	1847,767	ITGA5	Reactome_2022
6	Signaling By LRP5 Mutants R-HSA-5339717	1/6	0,003894	0,046096	0	0	333,0333	1847,767	DKK1	Reactome_2022
7	RUNX2 Regulates Genes Involved In Cell Migration R-HSA-8941332	1/8	0,005189	0,046096	0	0	237,8571	1251,416	ITGA5	Reactome_2022
8	POU5F1 (OCT4), SOX2, NANOG Repress Genes Related To Differentiation R-HSA-2892245	1/9	0,005836	0,046096	0	0	208,1146	1070,484	DKK1	Reactome_2022
9	Signaling By Activated Point Mutants Of FGFR1 R-HSA-1839122	1/11	0,007129	0,046096	0	0	166,475	822,9949	FGF5	Reactome_2022
10	FGFR3 Mutant Receptor Activation R-HSA-2033514	1/12	0,007774	0,046096	0	0	151,3333	735,0173	FGF5	Reactome_2022
11	Reversible Hydration Of Carbon Dioxide R-HSA-1475029	1/12	0,007774	0,046096	0	0	151,3333	735,0173	CA12	Reactome_2022
12	FGFR1c Ligand Binding And Activation R-HSA-190373	1/12	0,007774	0,046096	0	0	151,3333	735,0173	FGF5	Reactome_2022
13	FGFR2c Ligand Binding And Activation R-HSA-190375	1/13	0,00842	0,046096	0	0	138,7153	662,6705	FGF5	Reactome_2022
14	FGFR3 Ligand Binding And Activation R-HSA-190239	1/13	0,00842	0,046096	0	0	138,7153	662,6705	FGF5	Reactome_2022
15	FGFRL1 Modulation Of FGFR1 Signaling R-HSA-5658623	1/13	0,00842	0,046096	0	0	138,7153	662,6705	FGF5	Reactome_2022
16	Phospholipase C-mediated Cascade; FGFR3 R-HSA-5654227	1/13	0,00842	0,046096	0	0	138,7153	662,6705	FGF5	Reactome_2022
17	Synthesis Of Prostaglandins (PG) And Thromboxanes (TX) R-HSA-2162123	1/15	0,009709	0,046096	0	0	118,8869	551,0048	PTGES	Reactome_2022
18	Signaling By Activin R-HSA-1502540	1/15	0,009709	0,046096	0	0	118,8869	551,0048	FST	Reactome_2022
19	Negative Regulation Of TCF-dependent Signaling By WNT Ligand Antagonists R-HSA-3772470	1/15	0,009709	0,046096	0	0	118,8869	551,0048	DKK1	Reactome_2022
20	Phospholipase C-mediated Cascade: FGFR1 R-HSA-5654219	1/16	0,010353	0,046096	0	0	110,9556	507,1178	FGF5	Reactome_2022
21	FGFR1 Ligand Binding And Activation R-HSA-190242	1/16	0,010353	0,046096	0	0	110,9556	507,1178	FGF5	Reactome_2022
22	Formation Of Senescence-Associated Heterochromatin Foci (SAHF) R-HSA-2559584	1/17	0,010997	0,046096	0	0	104,0156	469,1243	HMGA2	Reactome_2022
23	Activated Point Mutants Of FGFR2 R-HSA-2033519	1/17	0,010997	0,046096	0	0	104,0156	469,1243	FGF5	Reactome_2022
24	SHC-mediated cascade:FGFR3 R-HSA-5654704	1/17	0,010997	0,046096	0	0	104,0156	469,1243	FGF5	Reactome_2022
25	PI-3K cascade:FGFR3 R-HSA-5654710	1/18	0,01164	0,046096	0	0	97,89216	435,9406	FGF5	Reactome_2022
26	Phospholipase C-mediated Cascade; FGFR2 R-HSA-5654221	1/18	0,01164	0,046096	0	0	97,89216	435,9406	FGF5	Reactome_2022
27	FRS-mediated FGFR3 Signaling R-HSA-5654706	1/19	0,012283	0,046096	0	0	92,44907	406,7303	FGF5	Reactome_2022
28	SHC-mediated cascade:FGFR1 R-HSA-5654688	1/20	0,012926	0,046096	0	0	87,57895	380,8382	FGF5	Reactome_2022
29	Signal Transduction By L1 R-HSA-445144	1/20	0,012926	0,046096	0	0	87,57895	380,8382	ITGA5	Reactome_2022
30	FGFR2 Ligand Binding And Activation R-HSA-190241	1/20	0,012926	0,046096	0	0	87,57895	380,8382	FGF5	Reactome_2022
31	PI-3K cascade:FGFR1 R-HSA-5654689	1/21	0,013568	0,046096	0	0	83,19583	357,7439	FGF5	Reactome_2022
32	Signaling By FGFR3 In Disease R-HSA-5655332	1/21	0,013568	0,046096	0	0	83,19583	357,7439	FGF5	Reactome_2022
33	FRS-mediated FGFR1 Signaling R-HSA-5654693	1/22	0,01421	0,046096	0	0	79,23016	337,0294	FGF5	Reactome_2022
34	SHC-mediated cascade:FGFR2 R-HSA-5654699	1/22	0,01421	0,046096	0	0	79,23016	337,0294	FGF5	Reactome_2022
35	PI-3K cascade:FGFR2 R-HSA-5654695	1/23	0,014852	0,046096	0	0	75,625	318,3547	FGF5	Reactome_2022
36	FRS-mediated FGFR2 Signaling R-HSA-5654700	1/24	0,015493	0,046096	0	0	72,33333	301,4412	FGF5	Reactome_2022
37	Downstream Signaling Of Activated FGFR3 R-HSA-5654708	1/24	0,015493	0,046096	0	0	72,33333	301,4412	FGF5	Reactome_2022
38	Signal Transduction R-HSA-162582	5/2465	0,015517	0,046096	0	0	4,452998	18,55029	FGF5;FOSI	Reactome_2022
39	Endodermal Cell Differentiation (GO:0035987)	2/31	0,000179	0,019946	0	0	125,1285	1079,321	HMGA2;IT	GO_Biological_Process_2023
40	Endoderm Formation (GO:0001706)	2/35	0,000229	0,019946	0	0	109,9394	921,3629	HMGA2;IT	GO_Biological_Process_2023
41	Negative Regulation Of Binding (GO:0051100)	2/86	0,001382	0,036266	0	0	43,08009	283,6409	HMGA2;DI	GO_Biological_Process_2023
42	Positive Regulation Of Macromolecule Metabolic Process (GO:0010604)	3/364	0,001493	0,036266	0	0	16,3097	106,1258	FGF5;HMC	GO_Biological_Process_2023
43	Negative Regulation Of Transmembrane Receptor Protein Serine/Threonine Kinase Signaling	2/111	0,002288	0,036266	0	0	33,15763	201,6045	FST;DKK1	GO_Biological_Process_2023
44	Positive Regulation Of Angiogenesis (GO:0045766)	2/119	0,002623	0,036266	0	0	30,87801	183,5178	HMGA2;IT	GO_Biological_Process_2023
45	Positive Regulation Of Wnt Signaling Pathway, Planar Cell Polarity Pathway (GO:2000096)	1/5	0,003246	0,036266	0	0	416,3125	2385,602	DKK1	GO_Biological_Process_2023
46	Cell Junction Disassembly (GO:0150146)	1/5	0,003246	0,036266	0	0	416,3125	2385,602	DKK1	GO_Biological_Process_2023
47	Regulation Of Monoatomic Anion Transport (GO:0044070)	1/5	0,003246	0,036266	0	0	416,3125	2385,602	STC1	GO_Biological_Process_2023
48	Positive Regulation Of Tau-Protein Kinase Activity (GO:1902949)	1/5	0,003246	0,036266	0	0	416,3125	2385,602	DKK1	GO_Biological_Process_2023
49	Negative Regulation Of Cardiac Muscle Cell Differentiation (GO:2000726)	1/5	0,003246	0,036266	0	0	416,3125	2385,602	DKK1	GO_Biological_Process_2023
50	Positive Regulation Of Gene Expression (GO:0010628)	3/480	0,003284	0,036266	0	0	12,27044	70,17205	FGF5;HMC	GO_Biological_Process_2023
51	Negative Regulation Of Apoptotic Process (GO:0043066)	3/482	0,003322	0,036266	0	0	12,21795	69,72839	HMGA2;IT	GO_Biological_Process_2023
52	Positive Regulation Of Cell Population Proliferation (GO:0008284)	3/483	0,003342	0,036266	0	0	12,19188	69,5082	FGF5;FOSI	GO_Biological_Process_2023
53	Positive Regulation Of Protein Kinase Activity (GO:0045860)	2/145	0,003864	0,036266	0	0	25,23077	140,1847	HMGA2;DI	GO_Biological_Process_2023
54	Negative Regulation Of Double-Strand Break Repair Via Nonhomologous End Joining (GO:200	1/6	0,003894	0,036266	0	0	333,0333	1847,767	HMGA2	GO_Biological_Process_2023
55	Negative Regulation Of Cardiocyte Differentiation (GO:1905208)	1/6	0,003894	0,036266	0	0	333,0333	1847,767	DKK1	GO_Biological_Process_2023
56	Positive Regulation Of Calcium Ion Import (GO:0090280)	1/7	0,004542	0,036266	0	0	277,5139	1497,033	STC1	GO_Biological_Process_2023
57	Regulation Of DNA-templated Transcription Initiation (GO:2000142)	1/8	0,005189	0,036266	0	0	237,8571	1251,416	FOSL1	GO_Biological_Process_2023
58	CD40 Signaling Pathway (GO:0023035)	1/8	0,005189	0,036266	0	0	237,8571	1251,416	ITGA5	GO_Biological_Process_2023
59	Synapse Pruning (GO:0098883)	1/8	0,005189	0,036266	0	0	237,8571	1251,416	DKK1	GO_Biological_Process_2023
60	Regulation Of Wnt Signaling Pathway, Planar Cell Polarity Pathway (GO:2000095)	1/8	0,005189	0,036266	0	0	237,8571	1251,416	DKK1	GO_Biological_Process_2023
61	Mesodermal Cell Differentiation (GO:0048333)	1/8	0,005189	0,036266	0	0	237,8571	1251,416	HMGA2	GO_Biological_Process_2023
62	Wound Healing, Spreading Of Epidermal Cells (GO:0035313)	1/9	0,005836	0,036266	0	0	208,1146	1070,484	ITGA5	GO_Biological_Process_2023
63	Regulation Of Actin Filament-Based Movement (GO:1903115)	1/9	0,005836	0,036266	0	0	208,1146	1070,484	STC1	GO_Biological_Process_2023
64	Regulation Of Tau-Protein Kinase Activity (GO:1902947)	1/9	0,005836	0,036266	0	0	208,1146	1070,484	DKK1	GO_Biological_Process_2023
65	Regulation Of Cardiac Muscle Cell Differentiation (GO:2000725)	1/9	0,005836	0,036266	0	0	208,1146	1070,484	DKK1	GO_Biological_Process_2023
66	Positive Regulation Of Vascular Endothelial Growth Factor Receptor Signaling Pathway (GO:0	1/9	0,005836	0,036266	0	0	208,1146	1070,484	ITGA5	GO_Biological_Process_2023
67	Negative Regulation Of Activin Receptor Signaling Pathway (GO:0032926)	1/10	0,006482	0,037598	0	0	184,9815	932,0597	FST	GO_Biological_Process_2023
68	Positive Regulation Of Non-Canonical Wnt Signaling Pathway (GO:2000052)	1/10	0,006482	0,037598	0	0	184,9815	932,0597	DKK1	GO_Biological_Process_2023
69	Epithelial Cell Morphogenesis (GO:0003382)	1/12	0,007774	0,038778	0	0	151,3333	735,0173	STC1	GO_Biological_Process_2023
70	Negative Regulation By Host Of Viral Transcription (GO:0043922)	1/12	0,007774	0,038778	0	0	151,3333	735,0173	HMGA2	GO_Biological_Process_2023
71	Prostanoid Biosynthetic Process (GO:0046457)	1/14	0,009064	0,038778	0	0	128,0385	602,2149	PTGES	GO_Biological_Process_2023
72	Negative Regulation Of Single Stranded Viral RNA Replication Via Double Stranded DNA Inter	1/14	0,009064	0,038778	0	0	128,0385	602,2149	HMGA2	GO_Biological_Process_2023
73	Regulation Of Cell Fate Specification (GO:0042659)	1/14	0,009064	0,038778	0	0	128,0385	602,2149	DKK1	GO_Biological_Process_2023
74	Negative Regulation Of Multicellular Organismal Process (GO:0051241)	2/231	0,009527	0,038778	0	0	15,68718	73,00239	STC1;DKK1	GO_Biological_Process_2023
75	Regulation Of Activin Receptor Signaling Pathway (GO:0032925)	1/15	0,009709	0,038778	0	0	118,8869	551,0048	FST	GO_Biological_Process_2023
76	Positive Regulation Of Sprouting Angiogenesis (GO:1903672)	1/15	0,009709	0,038778	0	0	118,8869	551,0048	ITGA5	GO_Biological_Process_2023
77	Prostanoid Metabolic Process (GO:0006692)	1/16	0,010353	0,038778	0	0	110,9556	507,1178	PTGES	GO_Biological_Process_2023
78	Negative Regulation Of RNA Biosynthetic Process (GO:1902679)	1/16	0,010353	0,038778	0	0	110,9556	507,1178	HMGA2	GO_Biological_Process_2023
79	Positive Regulation Of Stem Cell Proliferation (GO:2000648)	1/16	0,010353	0,038778	0	0	110,9556	507,1178	HMGA2	GO_Biological_Process_2023
80	Regulation Of Stem Cell Proliferation (GO:0072091)	1/16	0,010353	0,038778	0	0	110,9556	507,1178	HMGA2	GO_Biological_Process_2023
81	Negative Regulation Of Anoikis (GO:2000811)	1/16	0,010353	0,038778	0	0	110,9556	507,1178	ITGA5	GO_Biological_Process_2023
82	Prostaglandin Biosynthetic Process (GO:0001516)	1/17	0,010997	0,038778	0	0	104,0156	469,1243	PTGES	GO_Biological_Process_2023
83	Negative Regulation Of Striated Muscle Cell Differentiation (GO:0051154)	1/17	0,010997	0,038778	0	0	104,0156	469,1243	DKK1	GO_Biological_Process_2023
84	Mesenchyme Development (GO:0060485)	1/17	0,010997	0,038778	0	0	104,0156	469,1243	HMGA2	GO_Biological_Process_2023
85	Regulation Of Single Stranded Viral RNA Replication Via Double Stranded DNA Intermediate (	1/17	0,010997	0,038778	0	0	104,0156	469,1243	HMGA2	GO_Biological_Process_2023
86	Negative Regulation Of Calcium Ion Transport (GO:0051926)	1/17	0,010997	0,038778	0	0	104,0156	469,1243	STC1	GO_Biological_Process_2023
87	Negative Regulation Of Cellular Senescence (GO:2000773)	1/17	0,010997	0,038778	0	0	104,0156	469,1243	HMGA2	GO_Biological_Process_2023
88	Negative Regulation Of Monoatomic Ion Transport (GO:0043271)	1/18	0,01164	0,038778	0	0	97,89216	435,9406	STC1	GO_Biological_Process_2023
89	Negative Regulation Of Transcription By RNA Polymerase II (GO:0000122)	3/763	0,011878	0,038778	0	0	7,589605	33,6455	FST;HMGA	GO_Biological_Process_2023
90	Regulation Of Cellular Response To Growth Factor Stimulus (GO:0090287)	1/19	0,012283	0,038778	0	0	92,44907	406,7303	FST	GO_Biological_Process_2023
91	Regulation Of Acute Inflammatory Response (GO:0002673)	1/19	0,012283	0,038778	0	0	92,44907	406,7303	PTGES	GO_Biological_Process_2023
92	Negative Regulation Of Peptidyl-Serine Phosphorylation (GO:0033137)	1/19	0,012283	0,038778	0	0	92,44907	406,7303	DKK1	GO_Biological_Process_2023
93	Mesoderm Formation (GO:0001707)	1/20	0,012926	0,038778	0	0	87,57895	380,8382	HMGA2	GO_Biological_Process_2023
94	Regulation Of Calcium Ion Import (GO:0090279)	1/20	0,012926	0,038778	0	0	87,57895	380,8382	STC1	GO_Biological_Process_2023
95	Sensory Perception Of Pain (GO:0019233)	1/20	0,012926	0,038778	0	0	87,57895	380,8382	PTGES	GO_Biological_Process_2023
96	Endothelial Cell Development (GO:0001885)	1/20	0,012926	0,038778	0	0	87,57895	380,8382	STC1	GO_Biological_Process_2023
97	Regulation Of Anoikis (GO:2000209)	1/21	0,013568	0,040015	0	0	83,19583	357,7439	ITGA5	GO_Biological_Process_2023
98	Regulation Of Vascular Endothelial Growth Factor Receptor Signaling Pathway (GO:0030947)	1/22	0,01421	0,041209	0	0	79,23016	337,0294	ITGA5	GO_Biological_Process_2023
99	Negative Regulation Of Ossification (GO:0030279)	1/24	0,015493	0,044192	0	0	72,33333	301,4412	DKK1	GO_Biological_Process_2023
100	Wound Healing, Spreading Of Cells (GO:0044319)	1/25	0,016133	0,044559	0	0	69,31597	286,0578	ITGA5	GO_Biological_Process_2023
101	Regulation Of Double-Strand Break Repair Via Nonhomologous End Joining (GO:2001032)	1/25	0,016133	0,044559	0	0	69,31597	286,0578	HMGA2	GO_Biological_Process_2023
102	Regulation Of Cardiac Muscle Cell Contraction (GO:0086004)	1/26	0,016774	0,045173	0	0	66,54	272,0119	STC1	GO_Biological_Process_2023
103	Regulation Of Monoatomic Ion Transport (GO:0043269)	1/28	0,018053	0,045173	0	0	61,60494	247,3091	STC1	GO_Biological_Process_2023
104	Limb Development (GO:0060173)	1/28	0,018053	0,045173	0	0	61,60494	247,3091	DKK1	GO_Biological_Process_2023
105	Hematopoietic Progenitor Cell Differentiation (GO:0002244)	1/28	0,018053	0,045173	0	0	61,60494	247,3091	FST	GO_Biological_Process_2023
106	Heterotypic Cell-Cell Adhesion (GO:0034113)	1/29	0,018692	0,045173	0	0	59,40179	236,398	ITGA5	GO_Biological_Process_2023
107	Chondrocyte Differentiation (GO:0002062)	1/29	0,018692	0,045173	0	0	59,40179	236,398	HMGA2	GO_Biological_Process_2023
108	Negative Regulation Of DNA Binding (GO:0043392)	1/29	0,018692	0,045173	0	0	59,40179	236,398	HMGA2	GO_Biological_Process_2023
109	Positive Regulation Of Calcium Ion Transport (GO:0051928)	1/29	0,018692	0,045173	0	0	59,40179	236,398	STC1	GO_Biological_Process_2023
110	Integrated Stress Response Signaling (GO:0140467)	1/29	0,018692	0,045173	0	0	59,40179	236,398	FOSL1	GO_Biological_Process_2023
111	Prostaglandin Metabolic Process (GO:0006693)	1/30	0,019331	0,045454	0	0	57,35057	226,3078	PTGES	GO_Biological_Process_2023
112	Positive Regulation Of JUN Kinase Activity (GO:0043507)	1/30	0,019331	0,045454	0	0	57,35057	226,3078	DKK1	GO_Biological_Process_2023
113	Regulation Of Transmembrane Receptor Protein Serine/Threonine Kinase Signaling Pathway	1/31	0,019969	0,046329	0	0	55,43611	216,9521	FST	GO_Biological_Process_2023
114	Heterochromatin Formation (GO:0031507)	1/32	0,020607	0,046793	0	0	53,64516	208,256	HMGA2	GO_Biological_Process_2023
115	Negative Regulation Of Double-Strand Break Repair (GO:2000780)	1/33	0,021245	0,046793	0	0	51,96615	200,1544	HMGA2	GO_Biological_Process_2023
116	Negative Regulation Of Epithelial Cell Migration (GO:0010633)	1/33	0,021245	0,046793	0	0	51,96615	200,1544	STC1	GO_Biological_Process_2023
117	Regulation Of Sprouting Angiogenesis (GO:1903670)	1/33	0,021245	0,046793	0	0	51,96615	200,1544	ITGA5	GO_Biological_Process_2023
118	Regulation Of Cellular Senescence (GO:2000772)	1/35	0,022519	0,048375	0	0	48,90441	185,5134	HMGA2	GO_Biological_Process_2023
119	Regulation Of Cardiac Muscle Contraction (GO:0055117)	1/35	0,022519	0,048375	0	0	48,90441	185,5134	STC1	GO_Biological_Process_2023
120	Regulation Of Receptor Internalization (GO:0002090)	1/36	0,023156	0,048455	0	0	47,50476	178,88	DKK1	GO_Biological_Process_2023
121	Base-Excision Repair (GO:0006284)	1/36	0,023156	0,048455	0	0	47,50476	178,88	HMGA2	GO_Biological_Process_2023
122	Negative Regulation Of Endothelial Cell Migration (GO:0010596)	1/37	0,023792	0,048455	0	0	46,18287	172,6508	STC1	GO_Biological_Process_2023
123	Regulation Of Ossification (GO:0030278)	1/37	0,023792	0,048455	0	0	46,18287	172,6508	DKK1	GO_Biological_Process_2023
124	Positive Regulation Of Protein Phosphorylation (GO:0001934)	2/377	0,024097	0,048455	0	0	9,508848	35,42698	FGF5;ITGA	GO_Biological_Process_2023
125	Negative Regulation Of Programmed Cell Death (GO:0043069)	2/381	0,024575	0,048455	0	0	9,406572	34,86086	HMGA2;DI	GO_Biological_Process_2023
126	Heterochromatin Organization (GO:0070828)	1/39	0,025063	0,048455	0	0	43,74781	161,2707	HMGA2	GO_Biological_Process_2023
127	Regulation Of Receptor-Mediated Endocytosis (GO:0048259)	1/39	0,025063	0,048455	0	0	43,74781	161,2707	DKK1	GO_Biological_Process_2023
128	Fibroblast Growth Factor Receptor Signaling Pathway (GO:0008543)	1/39	0,025063	0,048455	0	0	43,74781	161,2707	FGF5	GO_Biological_Process_2023
129	Negative Regulation Of DNA-templated Transcription (GO:0045892)	3/1025	0,026096	0,049802	0	0	5,567025	20,29713	FST;HMGA	GO_Biological_Process_2023
130	Stem Cell Differentiation (GO:0048863)	1/41	0,026332	0,049802	0	0	41,55625	151,1384	HMGA2	GO_Biological_Process_2023
131	Receptor Antagonist Activity (GO:0048019)	2/30	0,000168	0,006045	0	0	129,6039	1126,521	FST;DKK1	GO_Molecular_Function_2023
132	Growth Factor Activity (GO:0008083)	2/85	0,001351	0,020618	0	0	43,60131	288,0826	FGF5;DKK1	GO_Molecular_Function_2023
133	Growth Factor Receptor Binding (GO:0070851)	2/96	0,001718	0,020618	0	0	38,47776	244,9676	FGF5;ITGA	GO_Molecular_Function_2023
134	Minor Groove Of Adenine-Thymine-Rich DNA Binding (GO:0003680)	1/6	0,003894	0,025259	0	0	333,0333	1847,767	HMGA2	GO_Molecular_Function_2023
135	Vascular Endothelial Growth Factor Receptor 2 Binding (GO:0043184)	1/8	0,005189	0,025259	0	0	237,8571	1251,416	ITGA5	GO_Molecular_Function_2023
136	Glutathione Binding (GO:0043295)	1/9	0,005836	0,025259	0	0	208,1146	1070,484	PTGES	GO_Molecular_Function_2023
137	cAMP Response Element Binding (GO:0035497)	1/9	0,005836	0,025259	0	0	208,1146	1070,484	HMGA2	GO_Molecular_Function_2023
138	DNA-(apurinic Or Apyrimidinic Site) Endonuclease Activity (GO:0003906)	1/11	0,007129	0,025259	0	0	166,475	822,9949	HMGA2	GO_Molecular_Function_2023
139	C2H2 Zinc Finger Domain Binding (GO:0070742)	1/11	0,007129	0,025259	0	0	166,475	822,9949	HMGA2	GO_Molecular_Function_2023
140	Vascular Endothelial Growth Factor Receptor Binding (GO:0005172)	1/12	0,007774	0,025259	0	0	151,3333	735,0173	ITGA5	GO_Molecular_Function_2023
141	Platelet-Derived Growth Factor Receptor Binding (GO:0005161)	1/12	0,007774	0,025259	0	0	151,3333	735,0173	ITGA5	GO_Molecular_Function_2023
142	Carbonate Dehydratase Activity (GO:0004089)	1/13	0,00842	0,025259	0	0	138,7153	662,6705	CA12	GO_Molecular_Function_2023
143	DNA Binding, Bending (GO:0008301)	1/17	0,010997	0,030453	0	0	104,0156	469,1243	HMGA2	GO_Molecular_Function_2023
144	Low-Density Lipoprotein Particle Receptor Binding (GO:0050750)	1/22	0,01421	0,034104	0	0	79,23016	337,0294	DKK1	GO_Molecular_Function_2023
145	Fibroblast Growth Factor Receptor Binding (GO:0005104)	1/22	0,01421	0,034104	0	0	79,23016	337,0294	FGF5	GO_Molecular_Function_2023
146	Lipoprotein Particle Receptor Binding (GO:0070325)	1/27	0,017414	0,036106	0	0	63,97756	259,1414	DKK1	GO_Molecular_Function_2023
147	Receptor Ligand Activity (GO:0048018)	2/319	0,017614	0,036106	0	0	11,2819	45,5685	FGF5;DKK1	GO_Molecular_Function_2023
148	Glutathione Transferase Activity (GO:0004364)	1/28	0,018053	0,036106	0	0	61,60494	247,3091	PTGES	GO_Molecular_Function_2023
149	DNA Secondary Structure Binding (GO:0000217)	1/33	0,021245	0,039388	0	0	51,96615	200,1544	HMGA2	GO_Molecular_Function_2023
150	Nucleosomal DNA Binding (GO:0031492)	1/34	0,021882	0,039388	0	0	50,38889	192,5902	HMGA2	GO_Molecular_Function_2023
151	Pluripotent Stem Cell Differentiation Pathway WP2848	2/48	0,000433	0,019056	0	0	78,81818	610,5071	FST;DKK1	WikiPathway_2023_Human
152	ncRNAs Involved In Wnt Signaling In Hepatocellular Carcinoma WP4336	2/85	0,001351	0,019056	0	0	43,60131	288,0826	FOSL1;DKI	WikiPathway_2023_Human
153	Hair Follicle Development Cytodifferentiation Part 3 Of 3 WP2840	2/87	0,001414	0,019056	0	0	42,57112	279,3154	FST;DKK1	WikiPathway_2023_Human
154	lncRNA In Canonical Wnt Signaling And Colorectal Cancer WP4258	2/93	0,001614	0,019056	0	0	39,75225	255,5756	FOSL1;DKI	WikiPathway_2023_Human
155	CKAP4 Signaling Pathway Map WP5322	2/109	0,002207	0,019056	0	0	33,7808	206,6028	ITGA5;DKI	WikiPathway_2023_Human
156	Wnt Signaling WP428	2/114	0,002411	0,019056	0	0	32,26461	194,4816	FOSL1;DKI	WikiPathway_2023_Human
157	Pleural Mesothelioma WP5087	3/437	0,002517	0,019056	0	0	13,5159	80,88903	FGF5;FOSI	WikiPathway_2023_Human
158	Mesodermal Commitment Pathway WP2857	2/145	0,003864	0,025598	0	0	25,23077	140,1847	HMGA2;DI	WikiPathway_2023_Human
159	BMP2 WNT4 FOXO1 Pathway In Primary Endometrial Stromal Cell Differentiation WP3876	1/13	0,00842	0,040567	0	0	138,7153	662,6705	DKK1	WikiPathway_2023_Human
160	Mammary Gland Development Pathway Puberty Stage 2 Of 4 WP2814	1/13	0,00842	0,040567	0	0	138,7153	662,6705	FOSL1	WikiPathway_2023_Human
161	EDA Signaling In Hair Follicle Development WP3930	1/13	0,00842	0,040567	0	0	138,7153	662,6705	DKK1	WikiPathway_2023_Human
162	OSX And miRNAs In Tooth Development WP3971	1/15	0,009709	0,042882	0	0	118,8869	551,0048	DKK1	WikiPathway_2023_Human

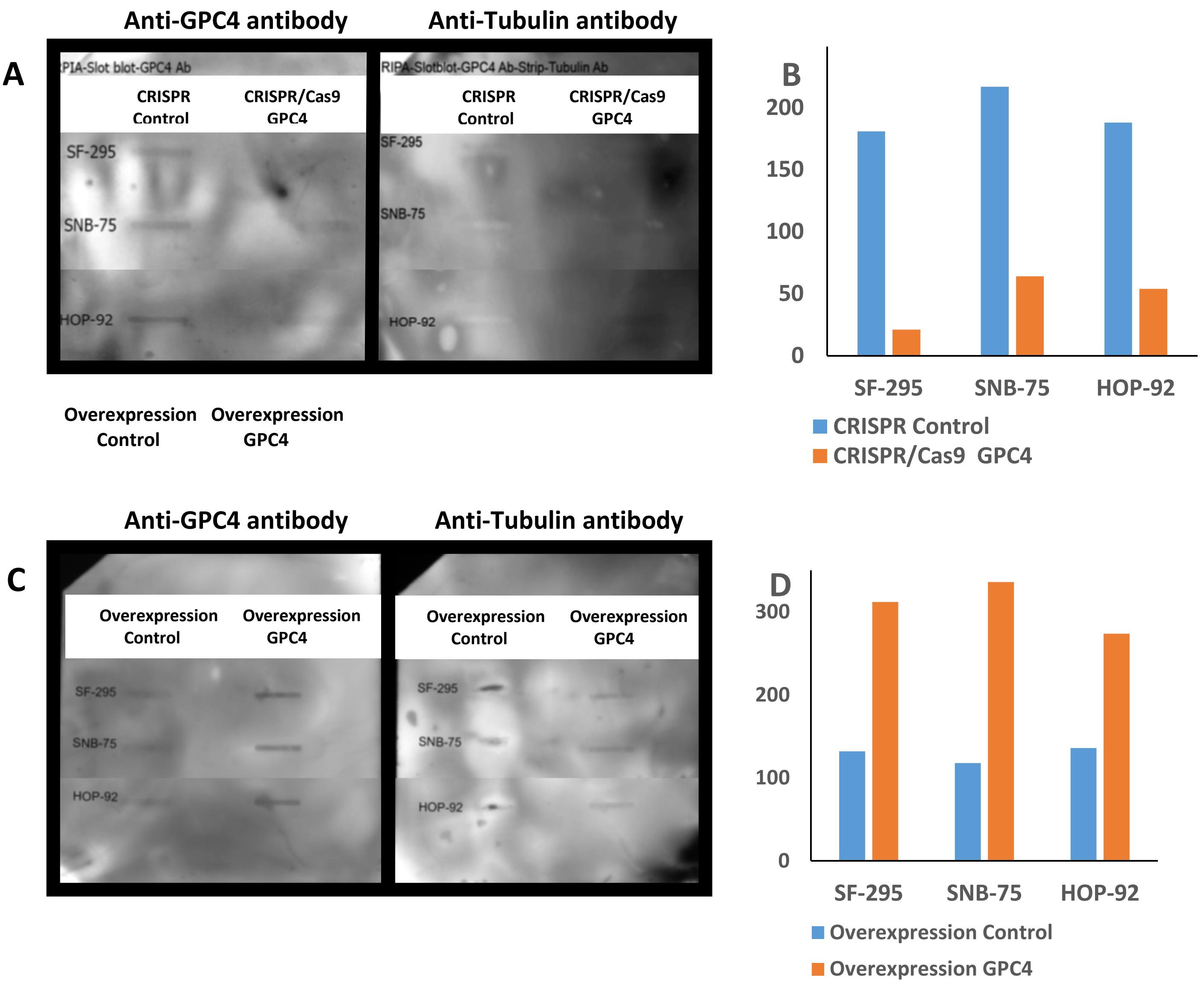

Supplementary Figure S1: Depletion or overexpression of GPC4 demonstrated by slot blot assay. (**A** and **B**) Depletion of GPC4 expression by CRISPR/Cas9 GPC4 or (**C** and **D**) overexpression of GPC4 by GPC4 overexpression vector in SNB-75, SF-295 and HOP-92 cells. Cells were transfected with (**A** and **B**) CRISPR control or CRISPR GPC4 vector or (**C** and **D**) overexpression control or GPC4 overexpression vector. Cell extracts were blotted onto PVDF membranes and probed against an anti-GPC4 primary antibody followed by a horseradish peroxidase-conjugated anti-rabbit IgG. Loading consistencies were determined by stripping the membranes and re-probing with an anti-α-tubulin antibody. In **B** and **D** staining intensities for GPC4 were recorded by densitometry measurements using GelAnalyzer 19.1. No signal was detected when the primary antibody was omitted.
